# Di-μ-chlorido-bis­[diacetonitrile­chlorido­oxidovanadium(IV)]

**DOI:** 10.1107/S1600536811037184

**Published:** 2011-09-17

**Authors:** Dalibor Dastych, Pavel Rotter, Gabriel Demo, Lenka Dastychová

**Affiliations:** a Department of Chemistry, Faculty of Technology, Tomas Bata University in Zlin, Nam. T. G. Masaryka 275 Zlin, 762 72, Czech Republic; b Department of Chemistry, Faculty of Science, Masaryk University, Kamenice 5 Brno–Bohunice, 625 00, Czech Republic

## Abstract

The title compound, [V_2_Cl_4_O_2_(CH_3_CN)_4_], is a centrosymmetric dinuclear V^IV^ complex associated with four mol­ecules of acetonitrile. The coordination around both V^IV^ atoms is essentially square-planar, involving three Cl atoms and one O atom [maximum deviation = 0.017 (3) Å for the O atom]. The augmented octahedral coordination of the metal atom is completed by the N atoms of acetonitrile ligands. The V^IV^ atoms are linked by two Cl atoms, acting as bridging atoms. The crystal studied was a non-merohedral twin with a ratio of the two twin components of 0.8200 (3):0.1800 (3). Although Cl and O atoms are present as potential acceptors in the title compound, no hydrogen bonds were observed in the crystal structure.

## Related literature

For the biological activity of vanadium(IV) compounds, see: D’Cruz *et al.* (2003 ▶); Lopez *et al.* (1976 ▶); Lu *et al.* (2001 ▶); Shi *et al.* (1996 ▶). For Ziegler–Natta catalysts, see: Hagen *et al.* (2002 ▶). For the synthesis of chloridooxidovanadium(IV) complexes, see: du Preez & Sadle (1967 ▶); Homden *et al.* (2009 ▶); Kern (1962 ▶); Papoutsakis *et al.* (2004 ▶); Priebsch & Rehder (1990 ▶).
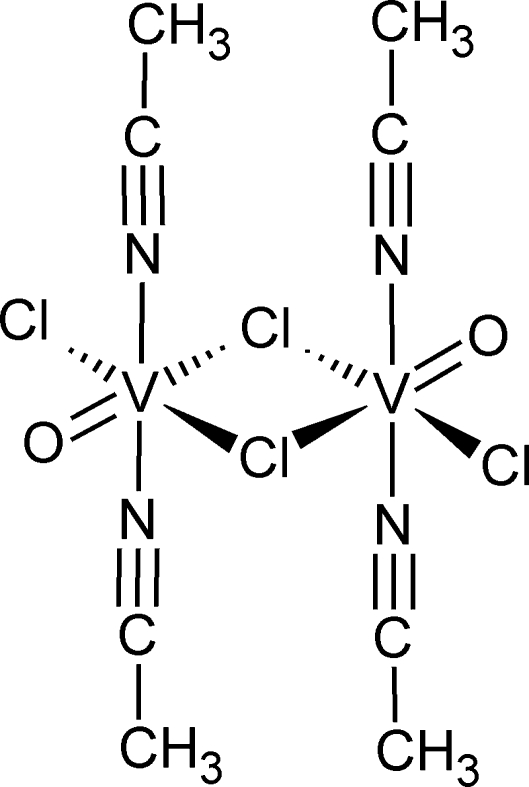


## Experimental

### 

#### Crystal data

[V_2_Cl_4_O_2_(C_2_H_3_N)_4_]
*M* *_r_* = 439.90
Triclinic, 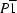

*a* = 7.0242 (6) Å
*b* = 8.1388 (6) Å
*c* = 8.7118 (5) Å
α = 86.536 (6)°
β = 66.806 (7)°
γ = 74.374 (7)°
*V* = 440.28 (6) Å^3^
*Z* = 1
Mo *K*α radiation
μ = 1.67 mm^−1^
*T* = 120 K
0.30 × 0.20 × 0.15 mm

#### Data collection

Oxford Diffraction Xcalibur Sapphire2 diffractometer
Absorption correction: multi-scan (*CrysAlis RED*; Oxford Diffraction, 2009 ▶) *T*_min_ = 0.804, *T*_max_ = 1.000
1550 measured reflections
1550 independent reflections
1432 reflections with *I* > 2σ(*I*)

#### Refinement

*R*[*F* ^2^ > 2σ(*F* ^2^)] = 0.031
*wR*(*F* ^2^) = 0.103
*S* = 1.25
1550 reflections
94 parameters
H-atom parameters constrained
Δρ_max_ = 0.47 e Å^−3^
Δρ_min_ = −0.51 e Å^−3^

### 

Data collection: *CrysAlis CCD* (Oxford Diffraction, 2009 ▶); cell refinement: *CrysAlis RED* (Oxford Diffraction, 2009 ▶); data reduction: *CrysAlis RED*; program(s) used to solve structure: *SHELXS97* (Sheldrick, 2008 ▶); program(s) used to refine structure: *SHELXL97* (Sheldrick, 2008 ▶) and *PLATON* (Spek, 2009 ▶); molecular graphics: *Mercury* (Macrae *et al.*, 2008 ▶); software used to prepare material for publication: *SHELXL97*.

## Supplementary Material

Crystal structure: contains datablock(s) I, global. DOI: 10.1107/S1600536811037184/ru2013sup1.cif

Structure factors: contains datablock(s) I. DOI: 10.1107/S1600536811037184/ru2013Isup2.hkl

Additional supplementary materials:  crystallographic information; 3D view; checkCIF report

## Figures and Tables

**Fig. 1.**
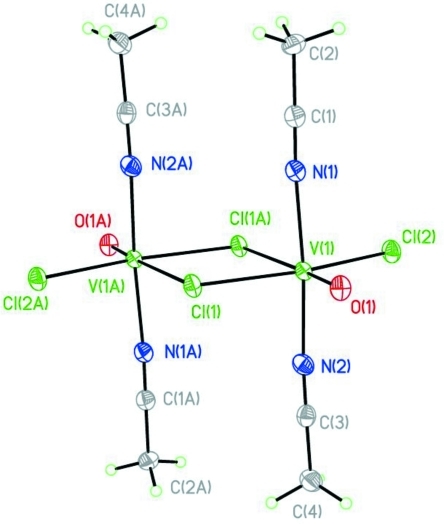
Crystal structure of the title compound. Thermal elipsoids are drawn with 50 % probability level, hydrogen atoms are represented as arbitrary spheres.

**Fig. 2.**
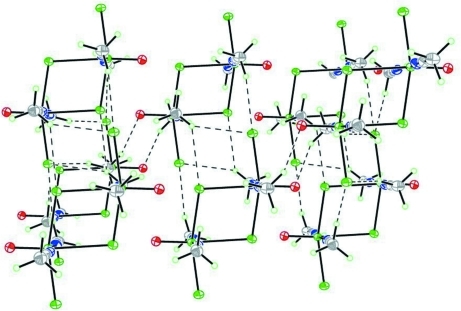
A view of the crystal structure of the title compound showing chains parallel to the ab-plane made up by C—H···Cl and C—H···O weak interactions (dashed lines). Thermal elipsoids are drawn with 50 % probability level.

## supplementary crystallographic information

### Comment

Vanadium(IV) compounds exert biological activity such as inhibition for some phosphatases (D'Cruz *et al.*, 2003; Lopez *et al.*, 1976), modulation of cell's redox potential (Lu  *et al.*, 2001) or catalysis of the generation of reactive oxygen species (Shi *et al.*, 1996). The oxovanadium(IV) complexes exhibit rapid selective spermicidal effects and their anti-HIV activity was studied too (D'Cruz *et al.*, 2003). Chlorovanadium(IV) compounds are also used for catalysis in homogenous Ziegler-Natta polymerizations to prepare high-molecular-weight polymers with narrow molecular weight distribution (Hagen *et al.*, 2002).

The dichloro(oxo)vanadium(IV) complex with acetonitrile was prepared for the first time by the reaction of VOCl_2_ with dry acetonitrile (du Preez *et al.* , 1967). The structure characteristic of the reaction product was performed only by means of UV, IR and conductivity measurements. The constitution of this reaction product was determined as VOCl_2_.2.5CH_3_CN. The only known crystal structure of acetonitrile adduct with dichloro(oxo)vanadium complex is to our knowledge [H_3_Np-tolyl][VOCl_3_(MeCN)_2_], which was prepared by the refluxing of [V(Np-tolyl)Cl_3_] in acetonitrile (Homden *et al.*, 2009).

It is known a lot of VOCl_2_ adducts with organic solvents, namely VOCl_2_.2THF (Kern, 1962) and trans-VOCl_2_(THF)_2_(H_2_O) (Papoutsakis *et al.*, 2004; Priebsch *et al.*, 1990), cis-VOCl_2_(CH_3_OH)_3_ (Papoutsakis *et al.*, 2004), trans-VOCl_2_(Et_2_O)_2_(H_2_O)_2_ (Papoutsakis *et al.*, 2004) or VOCl_2_(HMPA)_2_ (du Preez *et al.*, 1967). These adducts are presented in the known crystal structures as monomers in all cases (Papoutsakis *et al.*, 2004; Priebsch *et al.*, 1990). All of these complexes pick up very easily to the vanadium coordination sphere water molecules, therefore there are known only as water adducts (Papoutsakis *et al.*, 2004). On this account, it is necessary to keep strictly nonaqueous solution to obtain dichloro(oxo)vanadium complexes without water in the vanadium coordination sphere.

The asymmetric unit of the title compound consists of a single vanadium(IV) complex molecule associated with four molecules of acetonitrile (Fig. 1). Both of chlorine bridge atoms are situated essentially in the same plane with vanadium atoms, as demonstrated by torsion angles V1—Cl1—V1A—Cl1A 0.0° and O1—V1—Cl1—V1A, which is 179.36 (11)°, respectively. The angle describing the triple bond in acetonitrile is N2≡C3—C4 179.6 (4)° and N1≡C1—C2 179.1 (4)°, respectively. The crystal packing is showed in Fig. 2.

### Experimental

The title compound was obtained by the reaction of VOCl_3_ with *N*,*N'*-bis(trimethylsilyl)urea in acetonitrile. *N*,*N'*-bis(trimethylsilyl)urea (3.0 mmol) was dissolved in 100 cm^3^ of dry acetonitrile at 70 °C. The solutoin of VOCl_3_ (2.6 mmol) in in dry acetonitrile (50 cm^3^) was quickly added to the solution of *N*, *N'*-bis(trimethylsilyl)urea and the reaction mixture was refluxed for 17 h. The solvent was partially distilled off after the reaction and the total volume was reduced to 25 cm^3^. Dry CCl_4_ (25 cm^3^) was consequently added to the concentrated acetonitrile solution and two liquid phases were formed. Blue crystals of [(µ-Cl)_2_(VOCl_2_(CH_3_CN)_2_)_2_] grew up from the surface of the denser phase after 4 days standing at room temperature.

### Refinement

The investigated crystal was a non-merohedral twin [twin law: rotation of 180° around the [101] direction].. The twin law was determined using *TwinRotMat* implemented in *PLATON* (Spek, 2009). The twinning coefficient of the crystal is 0.180040. The description of twin law in transformation matrix is: (0.397 - 0.364 0.603) (0.000 - 1.000 0.000) (1.397 - 0.364 - 0.397) The detwinned data were obtained by HKLF 5 option in the *SHELXL97* program (Sheldrick, 2008) and the final refinement was carried out against the detwinned data set.

### Crystal data

**Table:**

[V_2_Cl_4_O_2_(C_2_H_3_N)_4_]	*Z* = 1
*M**_r_* = 439.90	*F*(000) = 218
Triclinic, *P*1	*D*_x_ = 1.659 Mg m^−^^3^
Hall symbol: -P 1	Mo *K*α radiation, λ = 0.7107 Å
*a* = 7.0242 (6) Å	Cell parameters from 5307 reflections
*b* = 8.1388 (6) Å	θ = 3.3–25.0°
*c* = 8.7118 (5) Å	µ = 1.67 mm^−^^1^
α = 86.536 (6)°	*T* = 120 K
β = 66.806 (7)°	Block, blue
γ = 74.374 (7)°	0.30 × 0.20 × 0.15 mm
*V* = 440.28 (6) Å^3^	

### Data collection

**Table:**

Oxford Diffraction Xcalibur Sapphire2 diffractometer	1550 independent reflections
Radiation source: Enhance (Mo) X-ray Source	1432 reflections with *I* > 2σ(*I*)
graphite	*R*_int_ = 0.000
Detector resolution: 8.4 pixels mm^-1^	θ_max_ = 25.0°, θ_min_ = 3.3°
ω scans	*h* = −7→8
Absorption correction: multi-scan (*CrysAlis RED*; Oxford Diffraction, 2009)	*k* = −9→9
*T*_min_ = 0.804, *T*_max_ = 1.000	*l* = −9→10
1550 measured reflections	

### Refinement

**Table:**

Refinement on *F*^2^	Primary atom site location: structure-invariant direct methods
Least-squares matrix: full	Secondary atom site location: difference Fourier map
*R*[*F*^2^ > 2σ(*F*^2^)] = 0.031	Hydrogen site location: inferred from neighbouring sites
*wR*(*F*^2^) = 0.103	H-atom parameters constrained
*S* = 1.25	*w* = 1/[σ^2^(*F*_o_^2^) + (0.0397*P*)^2^ + 1.0484*P*] where *P* = (*F*_o_^2^ + 2*F*_c_^2^)/3
1550 reflections	(Δ/σ)_max_ < 0.001
94 parameters	Δρ_max_ = 0.47 e Å^−^^3^
0 restraints	Δρ_min_ = −0.51 e Å^−^^3^

### Special details

**Table:**

Experimental. empirical absorption correction using spherical harmonics, implemented in SCALE3 ABSPACK scaling algorithm
Geometry. All e.s.d.'s (except the e.s.d. in the dihedral angle between two l.s. planes) are estimated using the full covariance matrix. The cell e.s.d.'s are taken into account individually in the estimation of e.s.d.'s in distances, angles and torsion angles; correlations between e.s.d.'s in cell parameters are only used when they are defined by crystal symmetry. An approximate (isotropic) treatment of cell e.s.d.'s is used for estimating e.s.d.'s involving l.s. planes.
Refinement. Refinement of *F*^2^ against ALL reflections. The weighted *R*-factor *wR* and goodness of fit *S* are based on *F*^2^, conventional *R*-factors *R* are based on *F*, with *F* set to zero for negative *F*^2^. The threshold expression of *F*^2^ > σ(*F*^2^) is used only for calculating *R*-factors(gt) *etc*. and is not relevant to the choice of reflections for refinement. *R*-factors based on *F*^2^ are statistically about twice as large as those based on *F*, and *R*- factors based on ALL data will be even larger.

### Fractional atomic coordinates and isotropic or equivalent isotropic displacement parameters (Å^2^)

**Table:**

	*x*	*y*	*z*	*U*_iso_*/*U*_eq_	
V1	0.58161 (10)	0.06353 (8)	0.67541 (8)	0.0141 (2)	
Cl1	0.71230 (14)	−0.15703 (11)	0.46017 (11)	0.0172 (2)	
Cl2	0.38000 (14)	0.30593 (12)	0.85537 (12)	0.0201 (2)	
O1	0.7642 (4)	−0.0069 (3)	0.7454 (3)	0.0192 (6)	
N2	0.7440 (5)	0.2175 (4)	0.5012 (4)	0.0197 (7)	
C4	0.9873 (6)	0.3945 (5)	0.3008 (5)	0.0228 (8)	
H4A	0.8981	0.5071	0.2916	0.034*	
H4B	1.0645	0.3351	0.1901	0.034*	
H4C	1.0911	0.4083	0.3448	0.034*	
C2	0.1588 (7)	−0.2589 (5)	1.0454 (5)	0.0241 (9)	
H2A	0.0118	−0.2274	1.0500	0.036*	
H2B	0.1555	−0.2385	1.1563	0.036*	
H2C	0.2258	−0.3801	1.0105	0.036*	
N1	0.3766 (5)	−0.0747 (4)	0.8326 (4)	0.0190 (7)	
C3	0.8514 (6)	0.2950 (5)	0.4132 (5)	0.0193 (8)	
C1	0.2821 (6)	−0.1565 (5)	0.9263 (5)	0.0190 (8)	

### Atomic displacement parameters (Å^2^)

**Table:**

	*U*^11^	*U*^22^	*U*^33^	*U*^12^	*U*^13^	*U*^23^
V1	0.0142 (3)	0.0150 (3)	0.0125 (3)	−0.0029 (2)	−0.0051 (3)	−0.0007 (2)
Cl1	0.0173 (4)	0.0172 (5)	0.0155 (5)	−0.0002 (3)	−0.0071 (4)	−0.0039 (3)
Cl2	0.0191 (5)	0.0192 (5)	0.0197 (5)	−0.0014 (4)	−0.0066 (4)	−0.0054 (4)
O1	0.0197 (14)	0.0202 (14)	0.0188 (14)	−0.0028 (11)	−0.0100 (11)	−0.0016 (11)
N2	0.0180 (16)	0.0195 (17)	0.0177 (17)	−0.0033 (14)	−0.0039 (14)	−0.0002 (14)
C4	0.025 (2)	0.024 (2)	0.020 (2)	−0.0099 (17)	−0.0079 (17)	0.0039 (16)
C2	0.025 (2)	0.024 (2)	0.023 (2)	−0.0136 (17)	−0.0040 (17)	0.0005 (17)
N1	0.0219 (16)	0.0209 (17)	0.0135 (16)	−0.0070 (14)	−0.0051 (14)	−0.0010 (14)
C3	0.0182 (19)	0.019 (2)	0.020 (2)	0.0008 (16)	−0.0096 (17)	−0.0040 (16)
C1	0.0200 (19)	0.0182 (19)	0.019 (2)	−0.0030 (16)	−0.0087 (16)	−0.0028 (16)

### Geometric parameters (Å, °)

**Table:**

V1—O1	1.588 (3)	C4—H4A	0.9800
V1—N1	2.085 (3)	C4—H4B	0.9800
V1—N2	2.086 (3)	C4—H4C	0.9800
V1—Cl2	2.3399 (10)	C2—C1	1.448 (6)
V1—Cl1	2.3969 (10)	C2—H2A	0.9800
V1—Cl1^i^	2.6836 (10)	C2—H2B	0.9800
Cl1—V1^i^	2.6836 (10)	C2—H2C	0.9800
N2—C3	1.139 (5)	N1—C1	1.138 (5)
C4—C3	1.453 (6)		
			
O1—V1—N1	94.79 (14)	C3—N2—V1	171.8 (3)
O1—V1—N2	95.52 (14)	C3—C4—H4A	109.5
N1—V1—N2	169.69 (13)	C3—C4—H4B	109.5
O1—V1—Cl2	99.62 (10)	H4A—C4—H4B	109.5
N1—V1—Cl2	89.60 (9)	C3—C4—H4C	109.5
N2—V1—Cl2	88.90 (9)	H4A—C4—H4C	109.5
O1—V1—Cl1	96.44 (10)	H4B—C4—H4C	109.5
N1—V1—Cl1	89.01 (9)	C1—C2—H2A	109.5
N2—V1—Cl1	89.61 (9)	C1—C2—H2B	109.5
Cl2—V1—Cl1	163.95 (4)	H2A—C2—H2B	109.5
O1—V1—Cl1^i^	174.92 (10)	C1—C2—H2C	109.5
N1—V1—Cl1^i^	84.57 (9)	H2A—C2—H2C	109.5
N2—V1—Cl1^i^	85.15 (9)	H2B—C2—H2C	109.5
Cl2—V1—Cl1^i^	85.42 (3)	C1—N1—V1	172.0 (3)
Cl1—V1—Cl1^i^	78.53 (4)	N2—C3—C4	179.6 (4)
V1—Cl1—V1^i^	101.47 (4)	N1—C1—C2	179.1 (4)
			
O1—V1—Cl1—V1^i^	179.36 (11)	Cl2—V1—Cl1—V1^i^	−0.47 (16)
N1—V1—Cl1—V1^i^	84.66 (9)	Cl1^i^—V1—Cl1—V1^i^	0.0
N2—V1—Cl1—V1^i^	−85.13 (9)		

Symmetry codes: (i) −*x*+1, −*y*, −*z*+1.
